# 含10天地西他滨预处理方案异基因造血干细胞移植治疗31例急性髓系白血病/骨髓增生异常综合征患者的疗效及安全性

**DOI:** 10.3760/cma.j.issn.0253-2727.2023.06.005

**Published:** 2023-06

**Authors:** 佳 刘, 易耕 曹, 荣莉 张, 卫华 翟, 欣 陈, 巧玲 马, 爱明 庞, 栋林 杨, 嘉璘 魏, 祎 何, 四洲 冯, 明哲 韩, 尔烈 姜

**Affiliations:** 1 中国医学科学院血液病医院（中国医学科学院血液学研究所），实验血液学国家重点实验室，国家血液系统疾病临床医学研究中心，细胞生态海河实验室，天津 300020 State Key Laboratory of Experimental Hematology, National Clinical Research Center for Blood Diseases, Haihe Laboratory of Cell Ecosystem, Institute of Hematology & Blood Diseases Hospital, Chinese Academy of Medical Sciences & Peking Union Medical College, Tianjin 300020, China; 2 天津医学健康研究院，天津 301600 Tianjin Institutes of Health Science, Tianjin 301600, China

**Keywords:** 地西他滨, 预处理, 急性髓系白血病, 骨髓增生异常综合征, 异基因造血干细胞移植, 10-day decitabine, Conditioning regimen, Acute myeloid leukemia, Myelodysplastic syndrome, Allogeneic hematopoietic stem cell transplantation

## Abstract

**目的:**

探讨含10天地西他滨预处理方案异基因造血干细胞移植（allo-HSCT）治疗急性髓系白血病（AML）/骨髓增生异常综合征（MDS）的早期疗效及安全性。

**方法:**

选择2021年4月至2022年5月间在中国医学科学院血液病医院接受含10天地西他滨（20 mg·m^−2^·d^−1^，−13 d～−4 d）预处理方案allo-HSCT的31例AML/MDS患者，对其临床资料进行分析。

**结果:**

①31例患者中男16例，女15例，中位年龄41（20～55）岁；AML 10例，MDS转变AML（MDS-AML）6例，慢性粒-单核细胞白血病转变AML（CMML-AML）1例，MDS 14例。②31例（100％）患者均获得粒细胞植入和血小板植入，中位植入时间分别为12（9～30）d、14（9～42）d。③预处理期间16例（51.6％）患者发生口腔黏膜炎，其中Ⅰ/Ⅱ级15例（48.4％），Ⅲ级1例（3.2％）；移植后13例（41.9％）患者发生巨细胞病毒血症，6例（19.4％）患者发生出血性膀胱炎，4例（12.9％）患者发生局部感染。④急性移植物抗宿主病（GVHD）中位发生时间为移植后33（12～111）d，移植后100 d急性GVHD累积发生率为41.9％（95％*CI* 26.9％～61.0％），Ⅲ/Ⅳ级急性GVHD累积发生率为22.9％（95％*CI* 13.5％～47.7％）；轻、中度慢性GVHD累积发生率为23.5％（95％*CI* 12.1％～43.6％），未发生重度慢性GVHD。⑤1例患者死于复发，未发生非复发死亡；移植后1年累积复发率为3.2％（95％*CI* 0.5％～20.7％）；移植后1年总生存率、无病生存率分别为92.9％（95％*CI* 80.3％～100.0％）、96.8％（95％*CI* 90.8％～100.0％）。

**结论:**

含10天地西他滨预处理方案allo-HSCT治疗AML/MDS可获得较好的早期疗效且安全性良好。

## Abstract (en)

**Objective:**

To investigate the early effect and safety of allogeneic hematopoietic stem cell transplantation（allo-HSCT）with a 10-day decitabine-containing conditioning regimen in the treatment of acute myeloid leukemia（AML）/myelodysplastic syndrome (MDS).

**Methods:**

From April 2021 to May 2022, 31 AML/MDS patients who received allo-HSCT with a 10-day decitabine-containing conditioning regimen were analyzed.

**Results:**

AML（*n*＝10）, MDS-AML（*n*＝6）, CMML-AML（*n*＝1）, and MDS（*n*＝14）were identified in 31 patients, 16 males, and 15 females, with a median age of 41（20–55）yr. Neutrophils and platelets were successfully implanted in 31 patients（100％）, with a median implantation duration of 12（9–30）and 14（9–42）days, respectively. During the preconditioning period, 16 patients（51.6％）developed oral mucositis, with 15 cases of Ⅰ/Ⅱ grade（48.4％）and one case of Ⅲ grade（3.2％）. After transplantation, 13 patients（41.9％）developed CMV viremia, six patients（19.4％）developed hemorrhagic cystitis, and four patients（12.9％）developed a local infection. The median time of acute graft versus host disease（aGVHD）following transplantation was 33（12–111）days. The cumulative incidence of aGVHD and Ⅲ/Ⅳ grade aGVHD was 41.9％（95％ *CI* 26.9％–61.0％）and 22.9％（95％ *CI* 13.5％–47.5％）, respectively. There was no severe cGVHD, and mild and moderate chronic GVHD（cGVHD）incidence was 23.5％（95％ *CI* 12.1％–43.6％）. As of November 30, 2022, only one of the 31 patients had relapsed, with a 1-yr cumulative relapse rate（CIR）of 3.2％（95％ *CI* 0.5％–20.7％）. There was only one relapse patient death and no non-relapse deaths. The 1-yr overall survival（OS）and disease-free survival（DFS）rates were 92.9％（95％ *CI* 80.3％–100％）and 96.8％（95％ *CI* 90.8％–100％）, respectively.

**Conclusion:**

A 10-day decitabine-containing conditioning regimen for allo-HSCT reduced relapse and was safe and feasible in treating AML/MDS.

异基因造血干细胞移植（allo-HSCT）目前仍是急性髓系白血病（AML）和骨髓增生异常综合征（MDS）的有效治疗手段。包含多种化疗药物/全身放射治疗（TBI）的移植前预处理是移植过程的关键环节[1]，其中以化疗为主的预处理方案在临床应用中效果显著[2]。随着新药物的加入，原发恶性疾病复发、移植相关死亡的发生率进一步降低，然而化疗药物相关的毒副作用以及后期引发的移植物抗宿主病（GVHD）等并发症仍是不可避免的[3]。此外，化疗对于消除残留的白血病细胞效果有限，而异体免疫细胞介导的移植物抗白血病（GVL）效应在根除肿瘤细胞、防治复发中具有关键作用[4]。因此，确定有效的预处理策略来减少复发，同时平衡甚至减轻移植相关不良反应、改善患者生存质量是改善AML/MDS移植患者预后的研究重点。

研究表明，去甲基化药物不仅可通过上调肿瘤细胞表面肿瘤抗原的表达来增强肿瘤特异性CD8^+^ T细胞的GVL效应，还可以通过上调调节性T细胞（Treg）表达来调节GVHD[5]–[6]。基于此，结合去甲基化药物地西他滨髓外毒性小以及在AML、MDS等血液肿瘤患者移植前治疗的有效性，不少学者将地西他滨加入至预处理方案中以期改善该类患者的移植预后[7]–[15]。本中心采用含5天地西他滨预处理方案allo-HSCT治疗44例MDS患者和4例慢性粒-单核细胞白血病（CMML）患者，获得了较好疗效，复发率为12％[16]。本中心采用含10天地西他滨预处理方案allo-HSCT治疗31例AML/MDS患者，以期在获得低复发率的同时进一步降低不良反应发生率，报告如下。

## 病例与方法

一、病例

本研究为前瞻队列研究，2021年4月至2022年5月期间于我院干细胞移植中心行allo-HSCT并符合以下条件的AML/MDS患者纳入本研究：①预后不良组AML患者（除外复发/难治患者）；②预后良好和预后中等组伴微小残留病（MRD）阳性AML患者；③修订的国际预后积分系统（IPSS-R）≥3.5分的MDS患者。AML和MDS的诊断分型参照世界卫生组织（WHO）2016诊断标准[17]，AML的遗传学分组参照2017欧洲白血病网（ELN）标准[18]；MDS的预后评估标准参照IPSS-R[19]。

二、预处理方案

移植前13 d开始地西他滨20 mg·m^−2^·d^−1^（−13 d～−4 d）；−9 d开始白消安（Bu）3.2 mg·kg^−1^·d^−1^× 3 d（−9 d～−7 d）；−6 d开始氟达拉滨（Flu）30 mg·m^−2^·d^−1^×3 d（−6 d～−4 d）和去氧柔红霉素（IDA）10 mg·m^−2^·d^−1^×3 d（−6 d～−4 d）；−3 d开始环磷酰胺（Cy）40 mg·m^−2^·d^−1^×2 d（−3 d、−2 d）。

三、GVHD的防治

GVHD的预防采用短疗程甲氨蝶呤（MTX）联合环孢素A（CsA）或他克莫司（FK506），单倍体移植患者加用霉酚酸酯（MMF）。同胞全相合移植且年龄>40岁的患者加用猪抗人淋巴细胞免疫球蛋白20 mg·kg^−1^·d^−1^×3 d，单倍体移植患者加用兔抗人胸腺细胞免疫球蛋白（rATG）2.5 mg·kg^−1^·d^−1^×4 d，无关供者移植患者加用rATG 2 mg·kg^−1^·d^−1^×4 d。

四、定义

AML复发定义为完全缓解（CR）后外周血重新出现白血病细胞或骨髓原始细胞占比>5％或发生髓外白血病细胞浸润。粒细胞植入：外周血中性粒细胞计数>0.5×10^9^/L连续3 d；血小板植入：外周血小板计数>20×10^9^/L连续7 d且脱离血小板输注。急性GVHD（aGVHD）的诊断参照文献[20]，慢性GVHD（cGVHD）的诊断及分级参考文献[21]。采用不良事件通用术语评价标准（CTCAE）5.0版评价预处理方案不良反应。以短串联重复序列、性染色体荧光原位杂交判定植入情况。

五、随访

采用查阅门诊/住院病历及电话联系方式进行随访，截止时间为2022年11月30日。中位随访时间为281（191～583）d。

六、统计学处理

采用R4.0.4软件进行数据分析。计量资料以“中位数（范围）”表示，分类资料用“频数（百分比）”表示。采用Kaplan-Meier法计算总生存（OS）和无病生存（DFS）率，累积复发率（CIR）、非复发死亡率（NRM）和GVHD累积发生率的计算使用竞争风险模型，其中非复发死亡是复发的竞争风险事件，非GVHD死亡是GVHD的竞争风险事件。双侧*P*<0.05为差异具有统计学意义。

## 结果

一、病例资料

2021年4月至2022年5月期间共纳入31例符合入组条件的AML/MDS患者，其中男16例，女15例，中位年龄41（20～55）岁。疾病类型：AML 10例，MDS转变AML（MDS-AML）6例，CMML转变AML（CMML-AML）1例，MDS 14例。10例原发AML患者中，预后不良6例，预后中等、良好分别为3、1例（均在治疗过程中出现MRD转阳或一直未转阴）。MDS患者的IPSS-R评分均≥3.5分（中危4例，高危7例，极高危3例）。所有患者从确诊到移植的中位时间为152（62～1 331）d。卡氏评分100分的患者占89％，所有患者的造血干细胞移植共患病指数（HCI-CI）均<3分。同胞全相合移植13例（41.9％），单倍体移植18例（58％）。患者的具体临床特征和移植参数见表1。

二、造血重建

所有的患者的移植方式为回输粒细胞集落刺激因子动员的供者外周血造血干细胞，中位CD34^+^细胞输注量为4.2（2.0～12.0）×10^6^/kg，中位单个核细胞输注量为10.6（3.6～15.0）10^8^/kg。31例（100.0％）患者均获得粒细胞植入和血小板植入，中位植入时间分别为12（9～30）d、14（9～42）d。从预处理开始至造血重建，中位红细胞输注量为12（2～34）U，中位血小板输注量为5（0～16）U。所有患者移植后骨髓供者细胞嵌合度均>95％，达到完全嵌合。

三、预处理方案不良反应及移植相关并发症

21例（87.1％）患者在预处理后发生粒细胞缺乏伴发热，16例（51.6％）患者发生口腔黏膜炎，17例（54.8％）患者出现恶心呕吐，26例（83.8％）患者发生腹泻（详见表2）。移植相关并发症包括巨细胞病毒（CMV）血症13例（41.9％）、出血性膀胱炎6例（19.4％）、肺部感染2例（6.5％）、泌尿系统感染2例（6.5％）。

四、GVHD发生情况

31例患者中14例发生aGVHD，其中Ⅰ级2例，Ⅱ级4例，Ⅲ级8例，未发生Ⅳ级GVHD和GVHD相关死亡。aGVHD中位发生时间为移植后33（12～111）d，移植后100 d的aGVHD累积发生率为41.9％（95％*CI* 26.9％～ 61.0％），Ⅲ/Ⅳ级aGVHD累积发生率为22.9％（95％*CI* 13.5％～ 47.7％）。所有患者随访时间均超过6个月，31例患者中7例发生cGVHD（轻度3例，中度4例），中位发生时间为移植后170（107～238）d，移植后1年cGVHD累积发生率为23.5％（95％*CI* 12.1％～ 43.6％）。

单倍体移植、同胞全相合移植患者的aGVHD累积发生率分别为50.0％、38.4％（*P*＝0.511），Ⅲ/Ⅳ级aGVHD累积发生率分别为22.2％、30.7％（*P*＝0.618），差异均无统计学意义。以CsA为基础GVHD预防方案组的Ⅲ/Ⅳ级aGVHD发生率高于以FK506为基础GVHD预防方案组（66.1％对9.1％，*P*<0.05）。多因素分析显示，以CsA为基础的GVHD预防方案是发生Ⅲ/Ⅳ级aGVHD的独立危险因素（*P*＝0.009）（表3）。

**表1:** 31例接受含10天地西他滨预处理方案异基因造血干细胞移植AML/MDS患者的临床特征

临床特征	结果
性别［例（%）］	
男	16（51.6）
女	15（48.4）
年龄［岁，*M*（范围）］	41（20~55）
疾病类型［例（%）］	
AML	10（32.3）
MDS-AML	6（19.4）
CMML-AML	1（3.2）
MDS	14（45.1）
确诊至移植的时间［d，*M*（范围）］	152（62~1 331）
移植时骨髓原始细胞>5%［例（%）］	7（22.6）
Karnofsky评分［例（%）］	
100分	25（80.6）
90分	6（19.4）
HCI-CI评分［例（%）］	
0分	24（77.4）
1分	6（19.4）
2分	1（3.2）
供者性别［例（%）］	
男	22（71.0）
女	9（29.0）
供者年龄［例（%）］	
≥40岁	14（45.2）
<40岁	17（54.8）
供受者ABO血型相合类型［例（%）］	
相合	17（54.8）
主要不合	9（29.0）
次要不合	4（12.9）
完全不合	1（3.2）
GVHD预防［例（%）］	
FK506+ MTX	9（29.0）
CsA+ MTX	4（12.9）
FK506+ MTX+MMF	13（41.9）
CsA+ MTX+MMF	5（16.1）
CD34^+^细胞输注量［×10^6^/kg，*M*（范围）］	4.2（2.0~12.0）
单个核细胞输注量［×10^8^/kg，*M*（范围）］	10.6（3.6~15.0）
移植后维持治疗［例（%）］	
阿扎胞苷	10（32.3）
西达本胺	3（9.7）

注 AML：急性髓系白血病；MDS：骨髓增生异常综合征；MDS-AML：MDS转变AML；CMML-AML：慢性粒-单核细胞白血病转化急性髓系白血病；ELN：欧洲白血病网；IPSS-R：修订版国际预后积分系统；HCI-CI：造血干细胞移植共患病指数；GVHD：移植物抗宿主病；FK506：他克莫司；CsA：环孢素A；MTX：甲氨蝶呤；MMF：霉酚酸酯

五、复发和生存

至随访截止，31例患者中仅1例复发，1年CIR为3.2％（95％*CI* 0.5％～ 20.7％）。该例患者为MDS-AML患者，移植前疾病进展状态，移植后获得CR，移植后4.5个月疾病复发，给予供者淋巴细胞输注（DLI）联合维奈克拉和阿扎胞苷治疗，未获得缓解，最终因原发病死亡。至随访截止，未发生非复发死亡。移植后1年OS率为92.9％（95％*CI* 80.3％～100.0％），DFS率为96.8％（95％*CI* 90.8％～100.0％）。

**表2:** 31例接受含10天地西他滨预处理方案的常见不良反应［例（％）］

不良反应	结果
发热	27（87.1）
ATG相关发热	16（51.6）
感染	19（61.2）
局部感染	16（51.6）
菌血症	3（9.7）
口腔黏膜炎	16（51.6）
Ⅰ~Ⅱ级	15（48.4）
Ⅲ级	1（3.2）
恶心或呕吐	17（54.8）
Ⅰ级	13（41.9）
Ⅱ级	4（12.9）
GVHD	3（8.8）
腹泻	26（83.8）
Ⅰ~Ⅱ级	21（67.7）
Ⅲ级	5（16.1）
肝功能损害	15（48.4）
Ⅰ~Ⅱ级	13（41.9）
Ⅲ级	2（6.5）
肾功能损害	11（35.4）
Ⅰ级	10（32.3）
Ⅱ级	1（3.2）

注 ATG：抗胸腺细胞球蛋白；GVHD：移植物抗宿主病

**表3:** 接受含10天地西他滨预处理方案造血干细胞移植患者发生Ⅲ/Ⅳ级急性移植物抗宿主病（GVHD）影响因素的多因素分析

影响因素	*HR*（95%*CI*）	*P*值
以环孢素A为基础的GVHD防治方案	17.842（2.035~156.410）	0.009
同胞全相合移植	0.676（0.099~4.590）	0.690
供者年龄≥40岁	6.911（0.919~51.960）	0.060
女供男模式	10.349（0.948~112.901）	0.055
急性髓系白血病	0.615（0.074~5.070）	0.650

## 讨论

近年来有不少学者尝试在传统清髓预处理方案中加入地西他滨以降低移植后复发率进而改善AML/MDS患者的移植疗效。Zhang等[10]回顾性分析了53例接受包含5天地西他滨（20 mg·m^−2^·d^−1^）的强化BUCY2预处理方案和41例仅接受BUCY2预处理方案的高危MDS患者的移植结果，发现地西他滨组的3年CIR明显低于BUCY2组（20.2％对39.0％，*P*＝0.034），3年OS、DFS率均高于BUCY2组（70.2％对43.9％，51.1％对43.9％，*P*<0.05）。Li等[15]在接受allo-HSCT的中高危AML患者中也得到类似的结果，通过倾向性评分每组匹配了46例接受含5天地西他滨和不含地西他滨的改良BUCY预处理方案的AML患者，单倍体移植地西他滨组有更好的2年OS率（84.8％对58.2％，*P*＝0.047）和更低的2年CIR（17.9％对40.0％，*P*＝0.036）。此外，Tang等[13]用含5天地西他滨改良预处理方案allo-HSCT治疗高危AML患者，发现对于处于疾病活动状态的AML患者，地西他滨组的2年OS率和DFS率均优于对照组（80.7％对43.5％，*P*＝0.011；64.9％对39.2％，*P*＝0.024）。

鉴于地西他滨髓外毒性小，有学者在应用地西他滨治疗AML/MDS时将其用量延长至10 d以进一步提高疗效[22]–[24]，但目前仍缺乏含10天地西他滨预处理方案的研究。在一项多中心前瞻性Ⅱ期研究中，含10天地西他滨联合非清髓预处理方案allo-HSCT治疗46例预后不良老年AML患者（中位年龄为60岁），移植后1年CIR为23％，OS率为70％[9]。该结果与5天地西他滨的研究结果类似，但该研究患者整体年龄偏大且采用了非清髓预处理方案，间接证实了含10天地西他滨预处理方案的疗效。本研究所有MDS患者均为相对高危组，绝大多数AML患者为预后不良组，仅4例（12.9％）AML患者为低中危组，但这些患者移植前MRD反复阳性，仍有一定复发风险，因此本组患者整体肿瘤细胞负荷相对较高，复发风险较大。基于此，我们采用含10天地西他滨清髓预处理方案allo-HSCT对这些患者进行治疗，所有患者均获得造血重建，移植后1年CIR为3.2％，OS率为92.9％（95％*CI* 80.3％～100.0％），DFS率为96.8％（95％*CI* 90.8％～ 100.0％），优于本中心前期含5天地西他滨预处理方案结果（中位随访552 d，复发率12％，OS率86％），提示含10天地西他滨预处理方案在减低复发率方面是有效的。

虽然研究提示地西他滨可通过免疫调节机制减缓GVHD的发病[25]–[26]，然而实际临床应用中的结果尚有争议。部分研究发现预处理中包含地西他滨对于GVHD的发生没有显著影响[10],[13]，但也有研究表明含5天地西他滨预处理方案组的aGVHD发生率显著低于对照组[8]，还有研究发现含5天地西他滨预处理方案组cGVHD的发生率高于对照组[15]。本组病例Ⅲ/Ⅳ级aGVHD发生率为22.9％，与本中心早期含5天地西他滨预处理方案（23％）相比没有增加[16]，但Ⅲ/Ⅳ级aGVHD发生率仍偏高。研究表明去甲基化药物可通过去甲基化作用上调主要组织相容性Ⅰ类抗原表达[27]，笔者推测这可能引起供者T细胞的异体免疫增强，在减少复发的同时可能增加GVHD的发生率，因此在实际临床应用中地西他滨对GVHD的确切影响仍待进一步研究。此外，较高的Ⅲ/Ⅳ级aGVHD发生率还可能与本队列中年龄≥40岁供者占比较高（45.2％）有关，本研究中8例患者发生Ⅲ级aGVHD，其中7例患者的供者年龄大于40岁。有趣的是，在亚组分析中，我们发现在Ⅲ/Ⅳ级aGVHD发生率上，以FK506为基础的GVHD防治方案明显优于CsA，该结果与本中心早期的研究[28]和Huang等[29]的研究结果类似，也提示在进行含地西他滨的预处理方案allo-HSCT中，以他克莫司为基础的GVHD防治方案可能是更好的选择，相关的机制有待进一步探索。

本组病例口腔黏膜炎的发生率为51.5％，且大部分为Ⅰ、Ⅱ级，仅3.2％的患者为Ⅲ级，发生率和严重程度低于以往大部分清髓性预处理方案的报道[30]–[31]。此外，本组病例预处理期间不良反应绝大多数为Ⅰ～Ⅱ级，均在恰当的治疗后好转，未发生非复发死亡，提示本组病例所采用的含10天地西他滨预处方案具有良好的安全性。

综上，本研究结果初步显示含10天地西他滨的预处理方案allo-HSCT治疗AML/MDS可获得较好的近期疗效且安全性良好。本研究病例数相对较少、随访时间较短且后期部分患者接受了移植后维持治疗，上述结论有待进一步验证。
